# A Systematic Review of the Design and Applications of Antimicrobial Peptides in Wound Healing

**DOI:** 10.7759/cureus.58178

**Published:** 2024-04-13

**Authors:** Aqeel Ahmad, Javed M Khan, Altaf Bandy

**Affiliations:** 1 Department of Medical Biochemistry, College of Medicine, Shaqra University, Shaqra, SAU; 2 Department of Food Science and Nutrition, Faculty of Food and Agricultural Sciences, King Saud University, Riyadh, SAU; 3 Department of Community Medicine, College of Medicine, Shaqra University, Shaqra, SAU

**Keywords:** peptide-based antibiotics, safety and toxicity of antimicrobial peptides, wound healing mechanism of antimicrobial peptide, efficacy of antimicrobial peptides in wound healing, applications of antimicrobial peptides, design of antimicrobial peptides

## Abstract

The sources of antimicrobial peptides (AMPs), also known as peptide-based antibiotics, are diverse, such as plants, animals, microorganisms including human leukocytes, saliva, human defense peptides, and human sweat. These natural sources provide a rich variety of AMPs with unique characteristics and potential therapeutic applications, including wound-healing and antimicrobial properties. AMPs derived from these sources have shown promise in combating a wide range of pathogens, making them valuable targets for further research and potential clinical applications. The design of AMPs for wound healing involves a meticulous process of structurally optimizing peptides to possess a unique combination of antibacterial and wound-healing characteristics. This systematic review was produced to show the design and applications of AMPs in wound healing. The terms "antimicrobial peptides AND wound healing" were used to search for articles published between September 2023 and January 2010. In the search, we found a total of 12958 articles, of which 12898 were excluded, and the remaining 60 articles were chosen for further study. This systematic review underscores the potential of AMPs as valuable tools in infection control and wound healing, showcasing their versatility and effectiveness in combating a wide range of pathogens. Overall, AMPs in wound healing display a diverse mechanism of action, influencing the inflammatory response, encouraging tissue regeneration, and aiding tissue remodeling, along with strong antibacterial activity. Furthermore, this systematic review addresses AMP toxicity studies, which include rigorous in vitro and in vivo examinations to determine potential cytotoxic effects, systemic toxicity, and any adverse responses connected with its usage in wound-healing applications.

## Introduction and background

Antimicrobial peptides (AMPs), sometimes called peptide-based antibiotics, can be found in a range of natural sources, including plants, animals, and microbes [1]. Many plants contain AMPs in their seeds, leaves, and roots [2]. Common AMPs discovered in diverse plant species include defensins and cyclotides [3]. Frogs, invertebrates, and humans also manufacture AMPs as part of their immune defense mechanisms [4]. One well-known example is the cathelicidin peptide, which is present in the immune cells of many vertebrate species [5]. Microorganisms such as bacteria, fungi, and even viruses create AMPs to protect themselves against competing microorganisms [6]. Additionally, a number of AMPs that had strong antibacterial activity and little cellular toxicity were developed and described [7].

AMPs have broad-spectrum action, which means they may target a diverse variety of pathogens such as bacteria, viruses, fungi, and even cancer cells [8]. Second, AMPs have excellent specificity and selectivity, which allows them to successfully target infections while sparing host cells. Finally, AMPs have a minimal chance of becoming resistant. This is owing to their distinct method of action, which focuses on the cell membrane rather than particular cellular components or metabolic pathways [9].

AMPs exert their antimicrobial activity by interacting with microbial membranes, leading to membrane disruption and cell death [1]. The mechanism of action of AMPs involves initial electrostatic interactions between the cationic AMPs and the anionic components of microbial membranes, such as lipopolysaccharides and phospholipids. This interaction disrupts membrane integrity by forming pores or channels, causing leakage of cellular contents and ultimately leading to cell lysis [2]. Various models have been proposed to explain the mechanism of membrane permeabilization by AMPs. These include the toroidal pore model, where peptides and lipids form toroidal structures within the membrane, and the barrel-stave model, where peptides insert into the membrane to form transmembrane channels [2,3]. Additionally, the carpet model suggests that AMPs cover the membrane surface, leading to disruption, while the aggregated channel model proposes the formation of peptide-lipid complexes that penetrate the membrane [1-3].

It has been demonstrated that AMPs contribute to wound healing by enhancing a number of tissue repair processes. A vital component of the extracellular matrix that gives tissues structural support, collagen, may be synthesized, and fibroblasts and epithelial cells can proliferate when exposed to AMPs [6]. Additionally, these peptides have the ability to cause fibroblasts and epithelial cells to undergo mitosis, which is necessary for wound closure and tissue regeneration [2-3].

Moreover, it has been shown that AMPs contain immunomodulatory properties that aid in wound healing. They can trigger bacterial lysis, raise antibody levels, stimulate helper T cell proliferation, activate lymphocytes to destroy infected cells, and improve macrophage phagocytosis [3]. These immune-suppressive effects aid in the removal of infections and the reduction of inflammation, which speeds up the healing process of wounds [4]. AMPs' capacity to interact with microbial membranes, resulting in membrane permeabilization and cell lysis, is the mechanism by which they aid in wound healing. AMPs have the ability to interfere with microbial membranes, hence impeding the progress of infection and promoting tissue repair [1,5,6]. A further benefit of AMPs' antibacterial action is their ability to stave off wound infections, which can impede the healing process [1].

Throughout history, numerous treatments and substances have been utilized to help in wound healing. Some of the earliest approaches, which date back millennia, included natural compounds like honey, herbs, and plant extracts [2]. Antimicrobial medications have evolved over time as a consequence of advancements in medical research, and they have been imperative in the recovery of wounds. A substantial role in wound restoration is exercised by AMPs because of an array of characteristics [10]. These peptides are essential components of the defense mechanism of the body and exhibit a wide range of antibacterial action. Furthermore, AMPs have been shown to positively influence immunity, making them even more useful in the setting of wound healing [11]. In addition to their antibacterial activities, AMPs are anti-inflammatory and can stimulate tissue repair and regeneration. This eventually leads to superior wound-healing outcomes, such as lower infection rates, quicker wound closure, and increased tissue regeneration [12]. Because of these characteristics, these AMPs are known as ancient weapons to fight against bacterial infections [12].

Antibiotic resistance has serious repercussions, including extended sickness, greater healthcare expenditures, higher mortality rates, and the possible emergence of resistant organisms. Combating antibiotic resistance necessitates a broad strategy, including the creation of novel drugs. The numerous origins of AMPs, which include plants, animals, and microorganisms, emphasize their broad-spectrum effect and low risk of resistance development when compared to standard antibiotics. In this systematic review, we focus on wound-healing AMPs extracted from various sources as well as those that were developed. Furthermore, we shall talk about the effectiveness, toxicity, and mechanism of AMP in wound healing.

## Review

Methods

The Preferred Reporting Items for Systematic Reviews and Meta-Analyses (PRISMA) principles for new systematic reviews, as stated elsewhere, were followed in the preparation of this systematic review [13]. Two Internet databases, PubMed and Science Direct, were searched for literature on AMPs and their uses in wound healing. August 2023 marked the beginning of the bibliographical research, which gathered the articles and finished in September 2023. We gathered the papers from Science Direct and PubMed by using the following search terms: "antimicrobial peptides AND wound healing."

Inclusion Criteria

This study included publications published between September 2023 and January 2010. We picked peptides that had both antibacterial and wound-healing actions. We considered articles that reported in vitro or in vivo wound-healing studies. It should be noted that we only selected whole research publications.

Exclusion Criteria

Articles published before 2010 were excluded from this review. We excluded review articles, letters to the editor, editorials, correspondence, dissertations, and reports from our systematic review. Furthermore, we eliminated articles that were exclusively discussed in in silico experiments. We also deleted papers with peptides that did not exhibit antibacterial activity but showed wound-healing activity. Research publications that did not match the title or abstract of the systematic review articles were not included in the study. The details of the 60 research papers considered in the present review are emphasized in Table 1.

**Table 1:** Research articles selected for this study

Selected article references	Year	Peptide name	Sequence	In vitro/in vivo	Study model
Oudhoff et al., 2010 [14]	2010	Histatin-2	RKFHEKHHSHREFPFYGDYGSNYLYDN	In vitro	Gingival fibroblasts cells
		LL-37	LLGDFFRKSKEKIGKEFKRIVQRIKDFLRNLVPRTES		
Yin and Fu-Shin, 2010 [15]	2010	LL-37	LLGDFFRKSKEKIGKEFKRIVQRIKDFLRNLVPRTES	In vitro	HCECs
Ramos et al., 2011 [16]	2011	P-LL37	PLLGDFFRKSKEKIGKEFKRIVQRIKDFLRNLVPRTES)	In vivo	Mice
		LL-37	LLGDFFRKSKEKIGKEFKRIVQRIKDFLRNLVPRTES		
Gibson et al., 2012 [17]	2012	hBD-3	GIINTLQKYYCRVRGGRCAVLSCLPKEEQIGKCSTRGRKCCRRKK	In vitro and in vivo	NIKS HKCs and murine
Williams et al., 2012) [18]	2012	Decapeptide (KSLW)	KKVVFWVKFK	In vivo	Mice
Huang et al., 2013 [19]	2013	Epi-1	FIFHIIKGLFHAGKMIHGLVTRRRH	In vivo	Mice
Chereddy et al., 2014 [20]	2014	LL-37	LLGDFFRKSKEKIGKEFKRIVQRIKDFLRNLVPRTES	In vitro	Keratinocytes
Gonzalez-Curiel et al., 2014 [21]	2014	HBD-2	GIGDPVTCLKSGAICHPVFCPRRYKQIGTCGLPGTKCCKKP	In vitro	Keratinocytes
		LL-37	LLGDFFRKSKEKIGKEFKRIVQRIKDFLRNLVPRTES		
Kim et al., 2014 [22]	2014	SHAP1	APKAMKLLKKLLKLQKKGI	In vitro and in vivo	HaCaT cell and mice
Tomioka et al., 2014 [23]	2014	SR-0379	MLKLIFLHRLKRMRKRLkRK	In vitro and in vivo	HUVECs and and NHDFs, rats
Björn et al., 2015 [24]	2015	PXL150		In vivo	Rats and rabbits
Huang et al., 2015 [25]	2015	TP3		In vivo	Mouse
Huang et al., 2015 [26]	2015	TP4		In vitro and in vivo	HaCaT, Hs-68, and mouse
Kasus-Jacobi et al., 2015 [27]	2015	Analog 120-146 WH		In vitro and in vivo	HCEC and mouse
Li et al., 2015 [28]	2015	AP-57	KRRPAKAWSGRRTRLCCHRVPSPNSTNLKGHHVRLCKPCKLEPEPRLWVVPGALPQV	In vivo	Rats
Silva et al., 2015 [29]	2015	LLKKK18	KLFKRIVKRILKFLRKLV	In vivo	Rats
Xie et al., 2015 [30]	2015	Temporin A		In vivo	Rats
Song et al., 2016 [31]	2016	Cys-KR12	CKRIVKRIKKWLR	In vitro	HaCaT and NHDF cells
Han et al., 2017 [32]	2017	Myxinidin2	KIKWILKYWKWS	In vivo	Mouse
		Myxinidin3	RIRWILRYWRWS		
Huang et al., 2017 [33]	2017	Epi-1	FIFHIIKGLFHAGKMIHGLVTRRRH	In vitro and in vivo	HaCaT cells and pigs
Liu et al., 2017 [34]	2017	B-2Ta		In vivo	Rats
Park et al., 2017 [35]	2017	Decapeptide (KSLW)	KKVVFWVKFK	In vitro	Gingival fibroblast
Cao et al., 2018 [36]	2018	cathelicidin-OA1	IGRDPTWSHLAASCLKCIFDDLPKTHN	In vitro and in vivo	HaCaT and mouse
Mi et al., 2018 [37]	2018	A-hBD-2	APKAMVTCLKSGAICHPVFCPRRYKQIGTCGLPGTKCCKKP	In vitro and in vivo	HaCaT cells and rats
Pfalzgraff et al., 2018 [38]	2018	Pep19-2.5	GCKKYRRFRWKFKGKFWFWG	In vitro and in vivo	HaCaT, HEK293 cells and female BALB/c mice
Saporito et al., 2018 [39]	2018	KR‐12	KRIVQRIKDFLR	In vitro	HaCaT cells
		VQ‐12V26	VQRIKVFLRNLV		
Wu et al., 2018 [40]	2018	Cathelicidin-NV	ARGKKECKDDRCRLLMKRGSFSYV	In vitro and in vivo	HaCat cells and mouse
Liu et al., 2014 [41]	2014	AH90	ATAWDFGPHGLLPIRPIRIRPLCG	In vivo	Murine
Lin et al., 2019 [42]	2019	AMP Tet213	KRWWKWWRRC	In vivo	Rats
Yang et al., 2019 [43]	2019	CAMP-A	LRRLKPLIRPWLRPLRRWWW	In vivo	Mice
		CAMP-B	RRRWRKRRWWW		
Shah et al., 2020 [44]	2020	Histatin-5		In vitro and in vivo	HCECs, HeLa cells, MCF-7 cells, and mice
Shi et al., 2020 [45]	2020	Cathelicidin-DM		In vivo	Mouse
Cheng et al., 2021 [46]	2021	Histatin-1	DSpHEKRHHGYRRKFHEKHHSHREFPFYGDYGSNYLYDN	In vitro and in vivo	3T3 cells and C57/BL6 male mice
Nagasundarapandian et al., 2021 [47]	2021	ΔPb-CATH4	TRSRWRRFIRGAGRFARRYGWRIA	In vivo	Mice
Suo et al., 2021 [48]	2021	KK(SLKL)_3_KK	KK(SLKL)_3_KK	In vivo	BALB/c female mice
Takahashi et al., 2021 [49]	2021	hBD-3	GIINTLQKYYCRVRGGRCAVLSCLPKEEQIGKCSTRGRKCCRRKK	In vitro and in vivo	Fibroblasts and male C57BL/6 mice
Fan et al., 2022 [50]	2022	Brevinin-2	GLMDSLKGLAATAGKTVLQGLLKTASCKLEKTC	In vitro	Human skin fibroblast cell
Farshadzadeh et al., 2022 [51]	2022	DCD-1L		In vivo	Mice
Huang et al., 2022 [52]	2022	NZ2114		In vitro and in vivo	BALB/c mice
Liu et al., 2022 [53]	2022	TP2-5	KKCIAKAILKKAKKLLKKLVNP	In vitro and in vivo	HaCaT cells, CCD-966SK Cells, HUVECs, and BALB/c mice
		TP2-6	KKCIAKAILKKAKKLLKDLVNP		
Rai et al., 2022 [54]	2022	LL-37	LLGDFFRKSKEKIGKEFKRIVQRIKDFLRNLVPRTESC	In vivo	Mice
Wang et al., 2022 [55]	2022	Cathelicidin-DM		In vitro and in vivo	HaCaT, HSF, HUVEC and RAW.264.7 cells and mice
Wu et al., 2022 [56]	2022	Pt5-1c		In vitro and in vivo	Fibroblasts and murine
Xu et al., 2022 [57]	2022	C-At5	CKIIKKIIKIIKKIIK-NH_2_	In vivo	Mouse
Yue et al., 2022 [58]	2022	AMP-IBP5	AVYLPNCDRKGFYKRKQCKPSR-NH2	In vivo	C57BL/6 mice
Zhou et al., 2022 [59]	2022	Jelleine-1	PFKLSLHL-NH_2_	In vitro and in vivo	HUVECs and rats
Cappiello et al., 2023 [60]	2023	Esc(1-21)	GIFSKLAGKKIKNLLISGLKG-NH_2_	In vitro and in vivo	hTCEpi cells and mouse
		Esc(1-21)-1c	GIFSKLAGKKIKNLLISGLKG-NH_2_		
Chen et al., 2023 [61]	2023	HX-12C	FFRKVLKLIRKIWR	In vivo	Rats
Gao et al., 2023 [62]	2023	RWPIL AMP	RWPIL	In vivo	Mice
Hu et al., 2023 [63]	2023	Esc-1a(1-21)NH_2_	GIFSKLAGKKIKNLLISGLKG-NH_2_	In vitro and in vivo	HUVECs and male BALB/c mice
Li et al., 2023 [64]	2023	C_8_G_2_	CH_3_(CH_2_)_6_C(O)-GIIKKIIKKI-NH_2_	In vitro and in vivo	Rat
Sen et al., 2023 [65]	2023	SP1V3_1	FLPIIKWVKKFFWRWR	In vitro and in vivo	NMDF cells and murine
Shi et al., 2023 [66]	2023	Chol-37(F34-R)	Chol-GLLSRLRDFLSDRGRRLGEKIERIGQKIKDLSERFQS	In vivo	Mice
Si et al., 2023 [67]	2023	Actinomycin X2	cyclic depsipeptide	In vivo	Rat
Soundrarajan et al., 2023 [68]	2023	Protegrin-1		In vitro and in vivo	HaCaT, IPEC-J2, KC, and mice
Yao et al., 2023 [69]	2023	AH-4	LKKWLKKWTLKASQFFGLM-NH_2_	In vivo	Mouse
Zhang et al., 2023 [70]	2023	KR-9	KPHAEVVLR	In vitro and in vivo	HGFs and rats
Zhang et al., 2023 [71]	2023	FWKFK	FWKFK	In vivo	Mice
Zheng et al., 2023 [72]	2023	*D*-GW1	(G(WIKK)_3_W)	In vivo	Rats
Zhou et al., 2023 [73]	2023	Jelleine-1	PFKLSLHL-NH_2_	In vivo	Mouse

Risk of Bias Assessment

The studies included in this systematic review were all from animal models or cell lines.

Results and discussion

**Figure 1:**
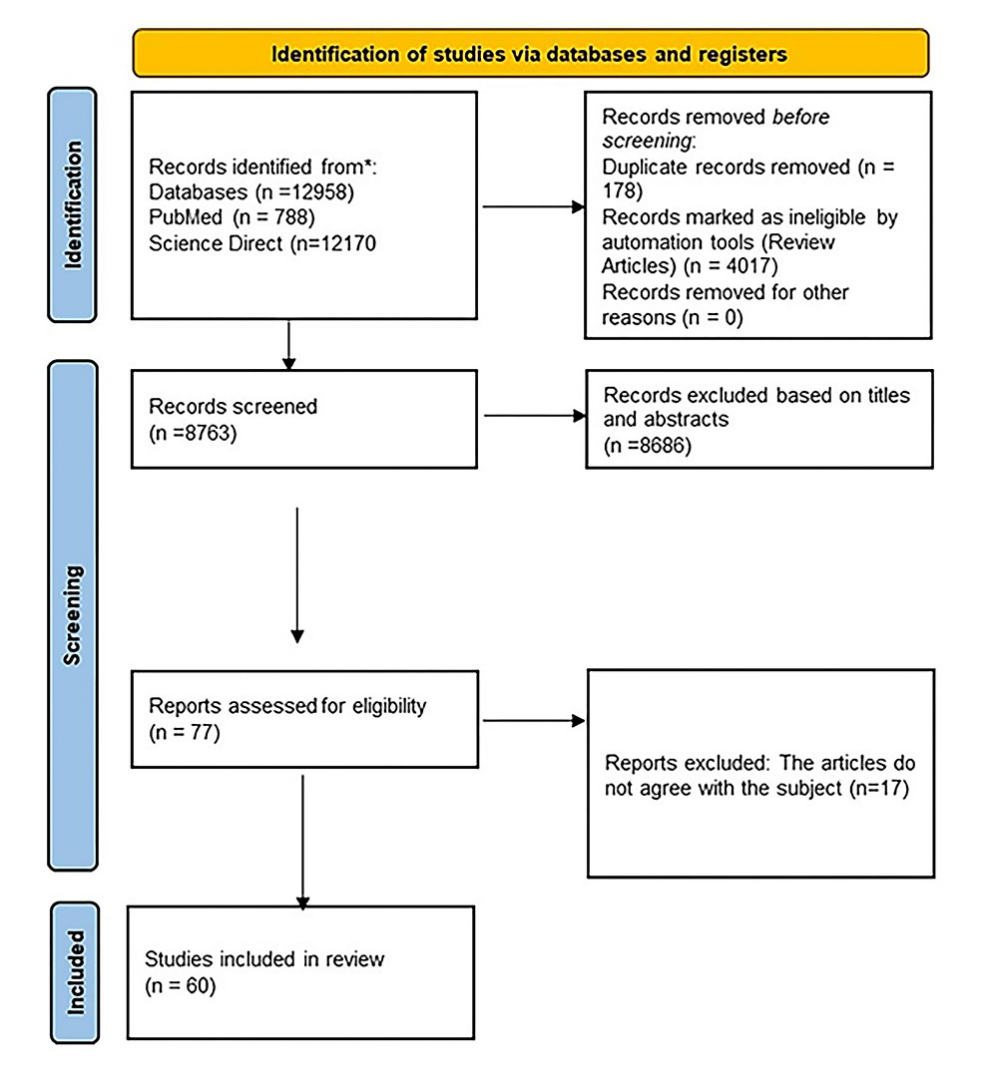
PRISMA 2020 flow diagram for systematic reviews, which includes searches of PubMed and Science Direct databases PRISMA: Preferred Reporting Items for Systematic Reviews and Meta-Analyses

A total of 12958 articles have been found in the database; of them, 788 were found in PubMed and 12170 in Science Direct. A total of 4017 review articles were eliminated from the analysis, and a total of 178 duplicate papers were eliminated. After recording the remaining 8763 articles, 8686 of them were disqualified according to their abstracts and titles. Ultimately, 77 publications were chosen for additional research, but 17 more articles were eliminated from the analysis as they had no clear connection to our systematic review article. So, 60 complete research publications were ultimately chosen for this systematic review (Table 1). Figure 1 shows the specifics of the article selection procedure.

Sources of Wound-Healing Antimicrobial Peptides

This section focuses on a range of wound-healing AMPs generated from diverse species. Human defense peptides (HDPs), including cathelicidins, defensins, and b-defensins (hBDs), have been discovered [14]. Marine organisms, particularly fish species and amphibians, are abundant sources of AMPs [33,41].

Human leukocytes, including neutrophils and epithelial cells, produce the AMP LL-37, which aids in wound healing [16]. Histatins, a kind of salivary AMP, have been isolated from the human parotid and submandibular glands [14]. Histatin-1, a wound-healing AMP, is mostly present in the human parotid and submandibular glands [14]. Histatin-5 was initially isolated from saliva and later found on ocular surfaces and tear films [44]. HDPs, notably cathelicidins and defensins, were identified [17]. Approximately six hBDs, numbered from one to six, have been isolated from humans [49]. Epinecidin-1 (Epi-1) has been found in marine organisms, including fish species [18,33]. DCD-1L is an anionic antibacterial isolated from human sweat [51]. Piscidins, an antibacterial peptide, were identified in fish mast cells. Tilapia piscidin 3 (TP3) and TP4 were discovered in a fish called Nile tilapia (*Oreochromis niloticus*) [25,26]. Another AMP, AP-57, has been isolated from human tissues, notably the digestive tract mucosa and skin epithelium [28].

Amphibians are an important source of AMPs, and numerous types of antimicrobials have been discovered with multifunctional capabilities. The European frog, *Pelophylax *kl.* esculentus* was used to extract brevinin-2Ta (B-2Ta) from the skin [34]. Cathelicidin-OA1, a distinct peptide, was discovered on the skin of the *Odorrana andersonii* frog [36]. Other AMPs, such as cathelicidin-NV, were isolated from the epidermis of the plateau frog, *Nanorana ventripunctata* [40]. AH90, a wound-healing AMP, was isolated from *Odorrana grahami*'s skin [41]. Cathelicidin-DM wound-healing AMP has been isolated from the toad *Duttaphrynus melanostictus* [45,55]. Brevinin-2 mature peptide (brevinin-2PN) was purified from a dark-spotted frog (*Pelophylax nigromaculatus*) [50]. Jelleine-1 was isolated from honeybee royal jelly [59,73]. The AMP Ac. The X2 strain is derived from *Streptomyces cyaneofuscatus*, a cyanobacterium identified from *Lyngbya* sp. [67]. Protegrin-1 was found in pig neutrophils [68]. KR-9 AMP was made from egg white [70].

Design of Wound-Healing Antimicrobial Peptides

Researchers have been focusing on developing AMPs with significant antibacterial properties that also help in wound healing. These peptides are designed to selectively target and disrupt bacteria's cell walls and membranes, allowing ions and ATPs to flow out [18]. Wound-healing AMPs also contain amino acid sequences that have been found to enhance cell migration, proliferation, and angiogenesis [16]. Because of their unique antibacterial and wound-healing properties, these peptides can effectively combat bacterial infections while also speeding up wound regeneration and repair [31]. Overall, the development of AMPs that promote wound healing while both targeting and eradicating microbial infections necessitates careful consideration of structural properties and amino acid sequences [24].

The bulk of antimicrobials utilized in wound healing are derived from naturally occurring AMPs. For example, a recombinant technique was employed to create P-LL37 from LL-37 as a template [16]. This designed peptide has a proline residue at the N terminus to maintain its immunophysiological properties in vitro and in vivo. This peptide promotes wound healing in mice by activating re-epithelialization and vascularization pathways [16]. Another LL-37 homolog, LLKKK18, was generated by substituting the original sequence's lysine residue with glutamine, asparagine, and aspartic acid [29]. LL-37 was utilized to create other peptides, including KR12 wound-healing AMP [31]. In addition, LL-37 pieces KR-12 and VQ-12V26 were constructed to disclose wound-healing activities [39].

The decapeptide KSL peptide was produced using combinatorial peptide libraries [18]. Later, a new peptide known as KSLW was generated by swapping Trp for Lys in the decapeptide KSL template [18,35]. SHAP1, an AMP that promotes wound healing, was designed with APKAM and LQKKGI at the N and C terminals, respectively [22]. In 2014, Tomioka et al. developed SR-0379, a 20-residue wound-healing AMP with anti-multidrug resistance capabilities that accelerated wound healing [23]. Björn et al. produced PXL150, a short-wound AMP with a broad spectrum and antibacterial efficacy against *Candida* spp. [24].

Analog 120-146 WH, a manmade peptide derived from the host defense peptide CAP37 [27], was developed to accelerate corneal wound healing. Myxinidin was the parent molecule for two smaller analogs, myxinidin2 and myxinidin3, which have wound-healing capabilities [32]. From hBD-2, the wound-healing AMP A-hBD-2 was developed [37]. Another synthetic peptide with wound-healing capabilities is Pep19-2.5 [38]. A new synthetic peptide, AMP Tet213, was developed and tested for its ability to stimulate wound healing [42].

Avian β-defensins AMPs were used to create two short CAMPs (CAMP-A and CAMP-B) [43]. Pb-CATH4 was converted into the wound-healing peptide ΔPb-CATH4 [47]. Furthermore, Suo et al. (2021) discovered KK(SLKL)3KK, a wound-healing AMP [48]. NZ2114 is a peptide produced from plectasin that shows strong wound-healing properties [52]. From tilapia piscidin, two wound-healing AMPs were developed, namely TP2-5 and TP2-6 [53]. Pt5 AMP, which is produced from zebrafish phosvitin, is the source of Pt5-1c, an AMP that aids in wound healing [56]. A synthetic AMP called C-At5 is created by adding a cysteine residue to the N terminus of At5 [57]. The synthetic AMP Esc(1-21) has 21 residues and is generated from Esculentin-1A, the parent AMP. Its diastereomer is Esc(1-21)-1c [60]. A unique synthetic AMP called HX-12C was created to hasten the healing of wounds [61]. A brief AMP was created for RWPIL, and it demonstrated effective wound healing [62]. C8G2 is a novel AMP that promotes wound healing [64]. Other newly discovered AMPs, such as SP1V3_1, were generated by adding the FLPII motif at the N-terminal of the snake venom cathelicidin, batroxicidin [65]. Chol-37(F34-R), a new wound-healing AMP, was developed utilizing PMAP-37 as a template [66]. In a similar vein, AMP Human hemokinin-1 served as the foundation for the creation of other AMPs, including AH-4 [69]. The novel AMP FWKFK was manufactured using Fmoc chemistry, and the product was tested using modified cell membrane chromatography [71]. D-GW1, a short AMP, was developed and has been shown to improve wound healing [72].

Efficacy of Antimicrobial Peptides in Wound Healing

This section examines the effectiveness of AMPs in wound healing, focusing on their function in encouraging various components of the healing process [21]. In vivo studies have shown that AMPs such as PXL150, TP3, and TP4 have microbicidal effects on pathogens, resulting in lower bacterial numbers and better wound healing. Furthermore, these AMPs have been demonstrated to inhibit pro-inflammatory cytokine activation at the infection site, indicating their possible role in wound healing [54]. Overall, AMPs have shown antimicrobial characteristics, increased fibroblast cell proliferation, angiogenesis, collagen deposition, and re-epithelialization, all of which aid in wound healing [15]. We have selected 60 publications to learn about their significance in wound healing, as indicated in Table 1. We looked at a number of AMPs from these 60 publications that demonstrated broad-spectrum antibacterial activations and hence contributed to wound healing.

It has been shown that LL-37 increases wound closure and airway epithelial cell proliferation, which aids in wound healing [21]. Furthermore, research has demonstrated that PLGA nanoparticles loaded with LL-37 promote angiogenesis and increase the production of VEGFa and IL-6, all of which benefit wound healing [20]. Furthermore, when immobilized in a wound dressing, LL-37 has been shown to speed up diabetic wound healing, proving its efficiency [54]. In an animal model of type II diabetes, the dressing was observed to accelerate wound healing in less than six days of contact; the dressing's bioactivity was predominantly mediated by tissue contact rather than LL37NPs leaking in the wound bed [54]. Furthermore, as proven, the peptide can up- or down-regulate cytokines to help in wound healing in the late phases of the healing process, demonstrating immunomodulatory properties [54]. According to another study, LL-37 promotes wound closure and epithelial cell proliferation, which aids in wound healing. In vitro studies have demonstrated that LL-37 phosphorylates the epidermal growth factor receptor (EGFR) in response to wounding and retains significantly higher amounts of phospho-EGFR, indicating that it may prolong EGFR signaling in response to wounding [15]. Furthermore, LL-37 has been shown to accelerate glucose-delayed corneal epithelial wound healing, indicating that it might be a beneficial therapy for wound closure [15].

According to the Ramos et al. study, LL-37 has been demonstrated to speed the healing process and encourage angiogenesis; after just seven days of therapy, re-epithelialization is nearly complete [16]. When applied topically, LL-37 improves control over the healing process and has been shown to nearly increase the number of endothelial cell-formed capillaries [16].

Some studies have shown that LL-37 peptide fragments can help in wound healing [39]. The study revealed that the LL-37 peptide segments KR-12 and VQ-12 V26 had a stronger effect on HaCaT cell migration, indicating their potential utility in wound healing [39]. Furthermore, because of their low cytotoxicity and combination of antibacterial and wound-healing properties, these peptides might be used as therapies to treat *Staphylococcus epidermidis* infections in human skin [39].

Due to the experiments, hBD-3 minimizes the methicillin-resistant strain of *Staphylococcus aureus* in burn wounds [17]. Takahashi et al. conveyed that, compared to wounds treated with a vehicle, wounds managed with hBD-3 exhibited notable indications of healing and recovered promptly [49]. The study additionally revealed that hBD-3 activates the FGFR/JAK2/STAT3 pathways, which in turn drive fibroblasts to migrate, multiply, and support angiogenesis [49].

According to certain studies, the AMP KSLW promotes wound healing by increasing fibroblast migration, collagen gel contraction, and reducing bacterial load [18,35]. KSLW improved in vitro fibroblast migration and collagen gel contraction, perhaps due to peptide-induced fibroblast-myo fibroblast transdifferentiation and increased α-SMA protein production [35]. The study found that combining Epi-1 and collagen in wounds improved healing, highlighting Epi-1's value in wound repair [19]. Furthermore, compared to normal and vancomycin-treated mice, the study revealed that animals treated with Epi-1 and Epi-1 + collagen exhibited faster wound closure [19].

The usefulness of histatin in wound healing is further supported by the observation that it promotes wound closure with both oral and non-oral cells [14]. SR-0379 has demonstrated efficacy in wound healing in a number of models. On days 8 and 15, SR-0379 significantly reduced the size of the unhealed lesion in an acute infection wound model, outperforming the usual therapy FGF2 [23]. Furthermore, in a streptozotocin-induced diabetic rat model, SR-0379 treatment resulted in a quick and significant fall in wound area on day 2, a continuous decrease in wound area on days 6 and 13, and complete wound healing by day 19 [23]. In a molecular sense, SR-0379 speeds up wound healing by activating the PI3K/Akt/mTOR pathway [23]. Furthermore, the peptide increased collagen production, granulation tissue formation, and tensile strength, indicating its efficacy in wound healing [23]. These findings suggest that SR-0379 might be an ideal medicine for treating burns, other incurable ulcers, and diabetic ulcers [23].

The research looked at the safety and effectiveness of the AMP PXL150 in treating burns that were infected [24]. PXL150 had a microbicidal action against *Pseudomonas aeruginosa* in vitro tests, and the HPC gel increased the antibacterial efficacy of PXL150 [24]. PXL150 effectively decreased bacterial counts in in vivo trials on mice with infected burn wounds, with notable results shown after just one day of therapy. Increased wound closure and reduced microbial loads in treated wounds have indicated that TP3 promotes the healing of infected wounds in mice [25]. Furthermore, TP3 therapy reduced the activation of pro-inflammatory cytokines TNF-α, IL-6, and CXCL5 at the infection site, indicating that it may have been used in the healing of wounds [25]. Sturdy antimicrobial activity was seen within 60 minutes of contact with TP4, which has been demonstrated to have antibacterial action both in vitro and in vivo [26]. Furthermore, it has been discovered that TP4 therapy decreases inflammatory cytokines at the infection site, including TNF and IL-6, and regulates epidermal healing by regulating fibroblast and keratinocyte proliferation and differentiation [26]. Additionally, it has been shown that TP4 therapy increases the gene expression of proteins that promote cell proliferation, including keratinocyte growth factor (KGF), collagen I, and collagen III, all of which are critical for wound healing [26].

It has been demonstrated that peptide 120-146 WH, which is produced from CAP37, is useful in hastening corneal wound healing in vivo and also decreasing bacterial infection in this infection area [27]. In addition to eradicating a *Pseudomonas aeruginosa* corneal infection in vivo, the peptide proved successful in encouraging re-epithelialization and healing in a corneal abrasion model [27]. Using a full-thickness excision model, the in vivo wound-healing efficacy of AP-57-NPs-H was investigated. The result suggested that the addition of this peptide accelerated wound healing and had almost full wound healing 14 days later [28].

A decrease in oxidative stress and inflammation brought about by LLKKK18 aided in quicker tissue repair and wound healing [29]. Furthermore, a quicker reduction in the size of the wound and appropriate collagen deposition with improved fiber synthesis in the granulation tissue were two further signs that LLKKK18 expedited the healing process [29]. Moreover, LLKKK18 increased the expression of vascular endothelial cadherin to enhance the integrity and stability of the newly created microvessels and caused a threefold increase in the density of the newly generated microvessels, indicating its pro-angiogenic action [29]. By controlling inflammation, lowering oxidative stress, quickening wound closure, encouraging appropriate collagen deposition, and stimulating angiogenesis, LLKKK18 has overall shown promise in wound healing [29]. In one study, temporin A-conjugated hydrogels dramatically reduced bacterial growth over the course of 24 hours when compared to negative controls, demonstrating the effectiveness of temporin A in wound healing [30]. Furthermore, temporin A-containing hydrogels demonstrated significantly reduced inflammation and granulation tissue development in an in vivo wound healing assessment utilizing a rat model with full-thickness wounds, suggesting a quicker wound-healing process [30]. These results imply that temporin A may be useful in accelerating wound healing and averting infections. Through encouraging HaCaT cell proliferation and differentiation as well as NHDF cell proliferation, the Cys-KR12-immobilized SF nanofiber membrane has shown effectiveness in wound healing [31]. Furthermore, it has demonstrated the capacity to suppress TNF-a expression in Raw264.7 cells, an essential function for quick wound healing [31]. These findings suggest that the SF nanofiber membrane immobilized with Cys-KR12 has excellent potential as a material for wound dressing [31].

**Figure 2:**
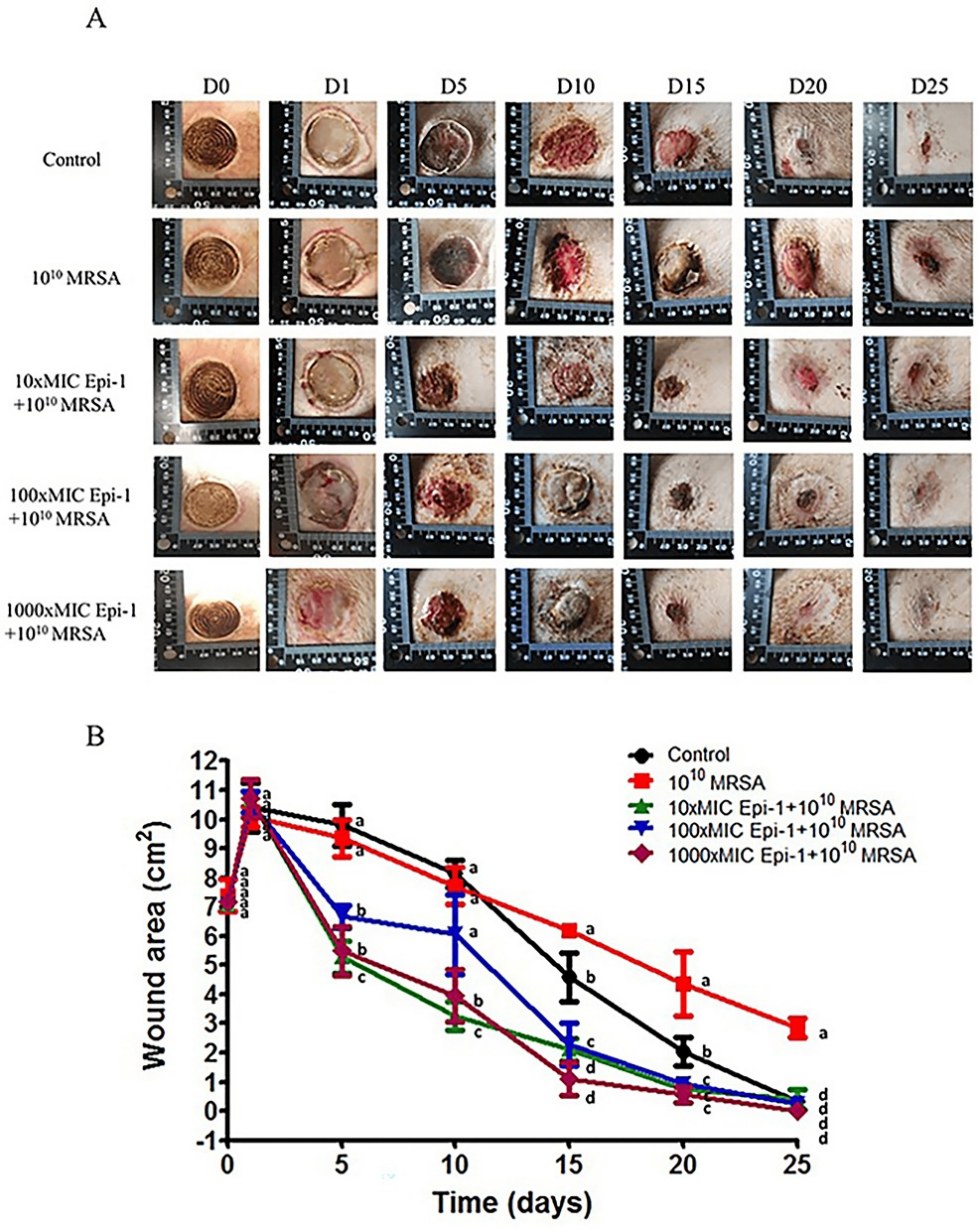
The effectiveness of AMP Epi-1 in treating MRSA-infected heat burn wounds in pigs: Panel A of the illustration demonstrates that Epi-1 totally healed the lesion after 25 days. Panel B depicts the size of the wound area from days 0 to 25 after Epi-1 therapy. Panel B demonstrates that the wound was totally healed following the delivery of 1000 MIC Epi-1 AMP: antimicrobial peptide, Epi-1: Epinecidin-1, MRSA: methicillin-resistant *Staphylococcus aureus* Image Credit: Adapted with permission from Huang et al., 2017 [33]

Myxinidin2 and myxinidin3 have shown efficacy in wound healing, particularly in infected wounds. In a mouse model, both peptides inhibited multidrug-resistant bacteria, enhanced wound healing, and completely healed the wound after some days [32]. Additionally, they stimulated EGFR phosphorylation and activation, promoting the migration of infected keratinocytes and ultimately aiding in wound closure [32]. By promoting the creation of extracellular matrix collagen surrounding the wound area and reducing sepsis, topical use of Epi-1 has been demonstrated to expedite the healing process (Figure 2) [33].

**Figure 3:**
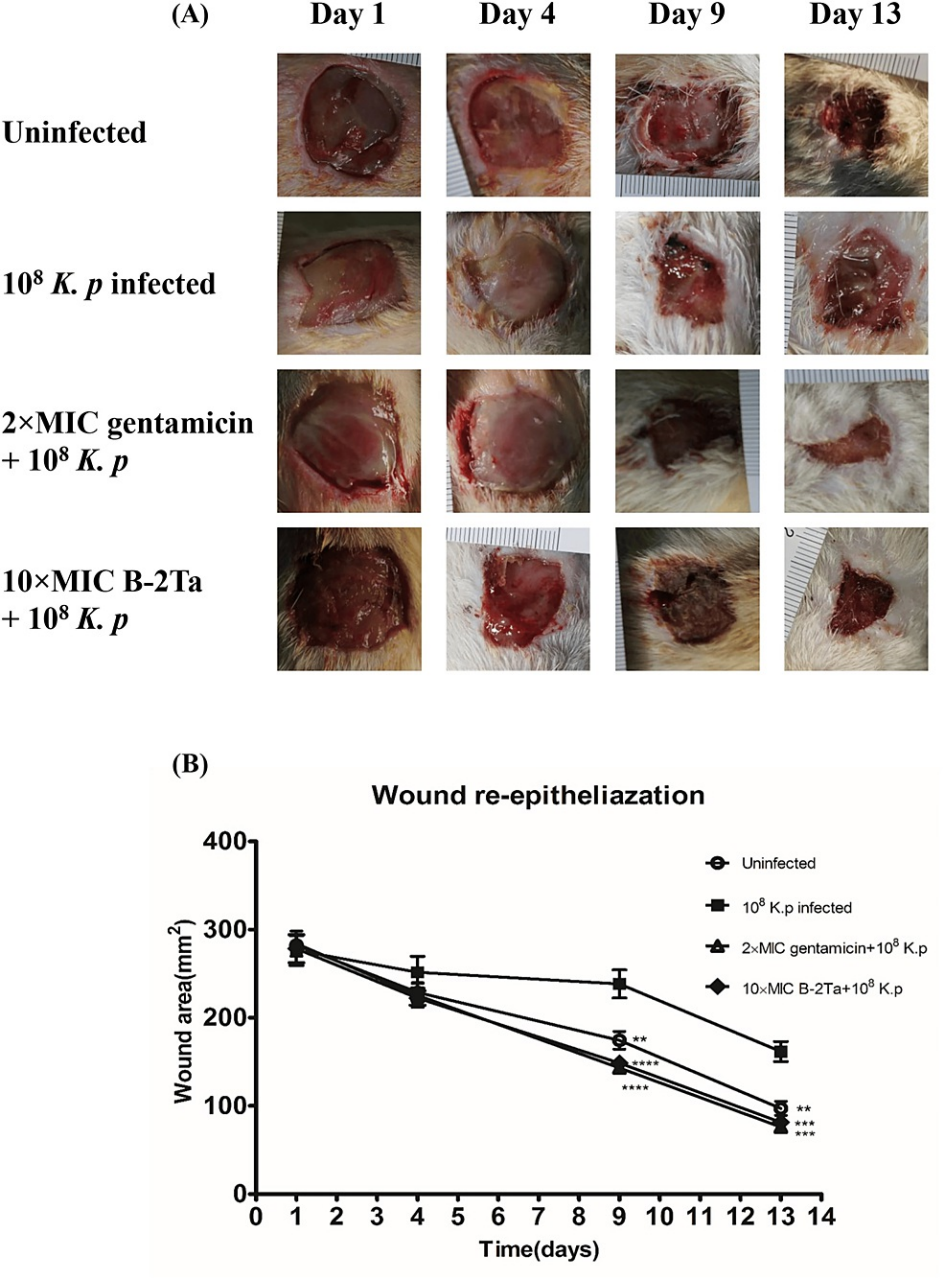
How well AMP B-2Ta worked to repair the wound of rats: Panel A displays the AMP B-2Ta wound-healing rate on days 1, 4, 9, and 13. Panel B depicts the wound area following AMP B-2Ta therapy over a period of 0–14 days AMP: antimicrobial peptide, B-2Ta: brevinin-2Ta Image Credit: Adapted with permission from Liu et al., 2017 [34]

When compared to the currently used curative antibiotic vancomycin, Epi-1 therapy has been validated to more effectively promote the production of collagen surrounding the wound site [33]. Moreover, it has been demonstrated that Epi-1 upsurges keratinocyte cell migration and proliferation in vitro and lowers methicillin-resistant *Staphylococcus aureus* (MRSA) numbers at the site of wound damage [33]. In a pig model, Epi-1 therapy enhanced vascularization and epithelial layer development [33]. The research findings indicate that at high concentrations, B-2Ta treatment significantly accelerated the rate of wound closure (Figure 3) [34].

Histopathological investigation of healing skins and morphometrical analysis of wound regions demonstrated time- and dose-dependent wound-healing activity following topical administration of cathelicidin-OA1 [36]. A different peptide produced from cathelicidin, called cathelicidin-NV, has demonstrated encouraging wound-healing properties. It is generated from the epidermal secretions of *Nanorana ventripunctata* [40]. It may aid in wound healing because it stimulates fibroblast differentiation, proliferation, collagen synthesis, and cytokine release [40]. The function of cathelicidin-NV in wound healing is further supported by the trigger of the ERK and p38 MAPK signaling pathways [40].

According to the study, A-hBD-2 showed better structural stability and more antibacterial activity than hBD-2, suggesting that it may be useful in the healing of wounds [37]. Additionally, it was demonstrated that A-hBD-2 stimulates keratinocyte migration and proliferation through the phosphorylation of STAT3 and EGFR, indicating its potential to facilitate wound healing [37]. Further evidence for the effectiveness of A-hBD-2 in wound healing comes from the fact that it caused wounds to close more quickly after treatment in Sprague-Dawley rats [37]. The synthetic peptide Pep19-2.5 is discussed in the study as a possible therapeutic tool for accelerating wound healing and reducing immunological and inflammatory responses in skin cells [38]. Additionally, using a mouse model, it investigates the wound-healing capabilities of Pep19-2.5 and demonstrates how it accelerates the pace of wound closure [38]. The P2X7 receptor, intracellular calcium, and reactive oxygen species (ROS) are all activated as part of the mechanism of action [38]. The discovery of AH90, a putative peptide that promotes wound healing, from the skin of the frog *Odorrana grahami* is discussed in this publication [41]. Additionally, AH90 promoted cell adhesion to laminin and fibronectin, which aided in the healing of wounds [41]. The research, both in vivo and in vitro, was used to assess the effectiveness of Tet213 in wound healing. The Tet213-modified dressing demonstrated strong antibacterial properties and enhanced NIH 3T3 fibroblast cell growth. Additionally, Tet213-conjugated wound dressing demonstrated enhanced angiogenesis, collagen deposition, and re-epithelialization, all of which support wound healing [42].

In addition to encouraging wound closure and preventing hepatic spread, CAMP-A dramatically decreased the bacterial burden in wounds [43]. The bacterial load was significantly decreased after five days of continuous application of CAMP-A to the infected lesion [43]. Furthermore, CAMP-A treatment on days 3 and 5 of the mouse trial promotes wound healing and induces the proliferation of epidermal cells [43]. Histatin-1 has been proven to efficiently stimulate wound healing, facilitate collagen deposition, and boost the number and activity of fibroblasts [46]. Additionally, it has been established that histatin-1 triggers the mTOR signaling pathway in fibroblasts, which is linked to the skin's broadened mechanical aspects and faster pace of wound healing [46]. In a mouse model of corneal damage, Hst5 has been demonstrated to greatly improve wound-healing rates [44]. Histological examination of the injured corneas revealed fewer corneal wounds in the Hst5-treated condition [44]. Hst5 has also been reported to improve cell spreading and encourage epithelial cell migration [44]. Furthermore, pro-migratory actions of Hst5 have been linked to ERK activation/phosphorylation, which suggests that cellular signaling pathways play a part in its wound-healing capacities [44].

Furthermore, in the mouse wound infection model, cathelicidin-DM has shown high therapeutic potential, suggesting that it might be used as a model for the development of antimicrobial drugs [45]. According to the study, cathelicidin-DM-treated wounds healed marginally quicker than those treated with gentamycin and control groups; a significant difference was seen two days after treatment [45]. According to different research, cathelicidin-DM has the ability to repair wounds and trigger the MAPK signaling pathway, which aids in the healing of skin wounds [55]. It encourages tissue re-epithelialization and granulation tissue development and speeds up entire skin wound healing in mice, even in infected wounds. Furthermore, cathelicidin-DM promotes collagen I deposition and α-smooth muscle actin expression, suggesting its function in the remodeling stage of wound healing [55]. Several tests were conducted to illustrate the effectiveness of ΔPb-CATH4 in wound healing. In an in vivo investigation, it was discovered that ΔPb-CATH4 therapy produced neo-epithelialization that resembled that seen with gentamicin treatment [47]. Moreover, mice treated with ΔPb-CATH4 showed full recovery and complete wound closure on day 14, demonstrating its efficiency in wound healing, according to the in vivo effects of the compound on wound closure following *Staphylococcus aureus* infection [47].

It was revealed that the AMP KK(SLKL)3KK was effective in speeding wound healing [48]. In comparison to the control and AMP groups, wounds treated with the AMP-HA hydrogel, which included KK(SLKL)3KK, demonstrated enhanced wound-healing manifestations [48]. On day five, the AMP-HA hydrogel group had the lowest wound area to its original size, and by day nine, the wound size had shrunk significantly, leading to complete healing by day 13 [48]. Furthermore, the AMP-HA hydrogel outperformed AMP alone in accelerating infected wound healing by increasing tissue re-epithelialization, angiogenesis, and collagen deposition [48]. The skin of the dark-spotted frog was employed in the study to find brevinin-PN, a new antibacterial peptide [48]. This peptide displayed remarkable wound-healing activity by speeding up the repair of scratches in human skin fibroblast cells and boosting growth factor gene expression [48].

**Figure 4:**
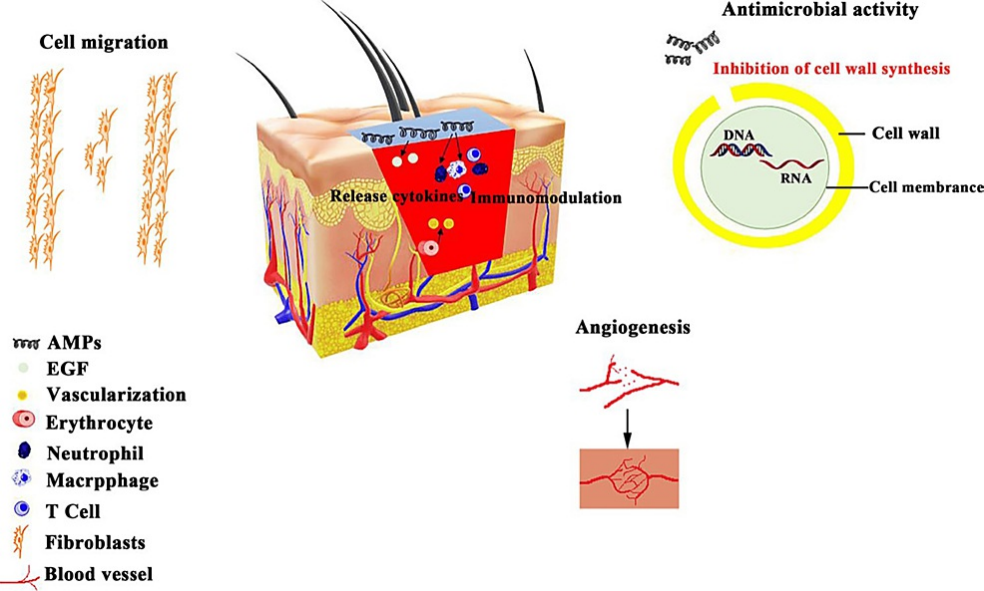
The mechanism of NZ2114 hydrogel in wound healing. NZ2114 hydrogel promotes cell migration, proliferation, and angiogenesis, which may speed up wound healing Image Credit: Adapted with permission from Huang et al., 2022 [52]

In vivo investigations that revealed that DCD-1L greatly reduced the bacteria in infected burn wounds provided evidence of the effectiveness of DCD-1L in wound healing [51]. According to the histopathological investigation, DCD-1L-induced wound healing was time-dependent, with marginal epithelium proliferation beginning on day five and ongoing re-epithelialization being seen on day ten [51]. The NZ2114 hydrogel is a viable choice for wound healing since it has shown better antibacterial activity than both ofloxacin and mupirocin [52]. Furthermore, it has been demonstrated that the NZ2114 hydrogel stimulates angiogenesis, cell migration, and proliferation, suggesting that it may hasten the healing of wounds (Figure 4) [52]. In addition, when compared to commercial medicines, the NZ2114-HPC hydrogel demonstrated superior antibacterial activity, decreased inflammation, and enhanced angiogenesis, underscoring its effectiveness in treating persistent skin injuries and drug-resistant *Staphylococcus aureus* bacterial infections [52].

Through a number of mechanisms, it has been established that TP2-5 and TP2-6 are effective in wound healing [53]. These chemicals promote collagen formation and KGF expression in fibroblasts, enhance keratinocyte and fibroblast migration and proliferation, and promote angiogenesis and endothelial cell migration [53]. According to these findings, TP2-5 and TP2-6 may help with wound healing by promoting tissue integrity and repair [53]. The designer peptide SHAP1 displayed wound-closing characteristics both in vitro and in vivo [22]. In vitro, SHAP1 enhanced HaCaT cell migration, and in vivo, it showed mild wound-healing activity [22].

Wu et al. (2022) found that Pt5-1c assists in the in vitro migration of both HELF and 3T3-L1 cells [56]. Additionally, this peptide promotes 3T3-L1 and HELF cell adhesion and proliferation [56]. Pt5-1c also induces collagen contraction, according to earlier in vitro studies [56]. When given topically to animal models, Pt5-1c has been found to promote wound healing [56]. AMP-IBP5 has been shown in both normal and diabetic mice to improve wound healing. When AMP-IBP5 was administered to wounds in normal mice, the wound area decreased significantly compared to when the vehicle was applied [58]. Furthermore, the effects of AMP-IBP5 were observed in diabetic mice on day 8, and by day 16, the wounds had completely healed, demonstrating the treatment's efficacy in hastening the process of delayed wound healing in diabetic mice [58].

The hydrogel made with Jelleine-1 and 8Br-cAMP was shown to have the ability to aid in wound healing both in vitro and in vivo [59]. Furthermore, the hydrogel had remarkable antibacterial activity and expedited the healing of diabetic wounds infected with MRSA, demonstrating the effectiveness of Jelleine-1 in managing bacterial infections in wound healing [59]. Further investigation by Zhou et al. (2023) proved the effectiveness of Jelleine-1 in wound healing [73]. The natural AMP Jelleine-1 created a peptide hydrogel that demonstrated strong antibacterial activity and superior biocompatibility. Notably, a mouse model with burn wounds infected with MRSA showed remarkable promise in accelerating wound healing [73].

Esc(1-21) and Esc(1-21)-1c were tested for their ability to promote wound healing using human corneal epithelial cells (HCECs) in an in vitro scratch assay [60]. The data revealed that both peptides helped close the gap area in a way that was dependent on time and dose. Esc(1-21) treatment produced significantly decreased scratch areas at 20 μM [60]. Furthermore, the investigation discovered that, rather than cell proliferation, cell migration is mostly responsible for the wound-healing activity caused by Esc peptides [60]. According to the measurement of the circularity index, the results demonstrated that Esc(1-21)-1c considerably enhanced corneal smoothness, suggesting that it may have the ability to promote wound healing [60]. The effectiveness of Esc(1-21)-1c in encouraging wound healing was further supported by the fluorescein staining results in mice, which showed that at certain doses, it considerably decreased the corneal epithelial wound area [60]. Esculentin-1a(1-21)A further research by Hu et al. (2023) found that NH2 accelerated wound healing by increasing collagen accumulation and angiogenesis, as evidenced by increased PCNA and CD31 expression [63]. Esculentin-1a(1-21)NH2's angiogenic activity was demonstrated in vitro by increasing cell migration and proliferation in HUVECs; this finding was connected to the activation of the PI3K/AKT pathway [63].

With a validated 98% inhibitory impact, the AMP HX-12C has proven to be very effective against bacteria, effectively reducing inflammation, and promoting wound healing [61]. The remarkable potential of the C8G2 peptide as a hydrogel dressing for the management of bacterially infected wounds is shown by the fact that it has been demonstrated to greatly speed the healing of skin wounds infected with MRSA [64]. The C8G2-containing hybrid hydrogel demonstrated a variety of properties, such as hemostasis, antimicrobial, and anti-inflammatory properties, in addition to encouraging tissue regeneration and cell migration [64]. Moreover, after 10 days, the wounds treated with BGA/C8G2 hydrogel nearly fully healed, demonstrating the hybrid hydrogel's ability to promote quick healing [64].

During a 24-hour test period, 10 μM SP1V3_1 indicated the most encouraging migratory propensity in scratch/migration testing utilizing dermal fibroblast cells when compared to untreated and ciprofloxacin controls [65]. SP1V3_1 showed a marked increase in healing in in vivo research conducted on albino Wistar rats [65]. Chol-37(F34-R) has demonstrated its ability to accelerate wound healing by acting as an antimicrobial [66]. Furthermore, mice treated with the antibacterial hydrogel containing Chol-37(F34-R) showed a notable improvement in the look of wound healing and better skin tissue appearance [66].

Actinomycin X2 (Ac. X2) is a viable option for wound-healing applications because of its good antibacterial and angiogenesis action [67]. Ac. X2-immobilized silk fibroin (SF) (ASF) film was produced by immobilizing Ac. X2 onto SF fibers. This film showed enduring antibacterial activity and decreased cytotoxicity, making it appropriate for wound-healing applications [67]. Moreover, Ac. X2 has demonstrated promise in accelerating wound healing and has been found to have stronger antibacterial activity than actinomycin D [67]. During wound healing, PG1 has been demonstrated to encourage cell migration and proliferation [68]. It has been shown that PG1 treatment causes intestinal porcine epithelial cells (IPEC-J2) and human KCs (HaCaT) to migrate more freely in a concentration-dependent manner [68]. Furthermore, it has been shown that PG1 causes KCs to migrate to aid in wound closure, as demonstrated by the scratch repair activity seen in KCs treated with PG1 [68].

In a variety of wound types, including diabetic and *Staphylococcus aureus*-infected animals, the effectiveness of AH-4 in wound healing has been shown. Significant wound-healing activity was shown by AH-4, and there was no discernible variation in effectiveness at varied doses [69]. AH-4 not only averted bacterial infection but also markedly boosted wound closure in a wound model infected with bacteria, unlike vancomycin, which only illustrated the antibacterial effect [69]. Furthermore, in contrast to the control group, AH-4 revealed a substantial rise in wound healing in the diabetic mouse model, implying that it has substantial therapeutic potential for the management of diabetic wound healing [69]. It has been demonstrated that in vitro migration and wound healing are significantly impacted by the peptide KR-9. Human gingival fibroblasts (HGFs) recovered at a rate about better than that of the control group after obtaining therapy with KR-9, and the number of migrating cells also rose [70]. This illustrates KR-9's potent ability to stimulate HGF migration, which was more notable than its ability to stimulate proliferation. Furthermore, KR-9 was shown to hasten the palatal healing process in rats with oral wounds, considerably decreasing the size and width of the lesion on days 7 and 11 following the procedure. Furthermore, the PI3K/AKT/mTOR signaling pathway is triggered as part of the mechanism by which KR-9 promotes wound healing [70].

Research showing that FWKFK significantly aided in the promotion of wound healing in mice provided evidence of its effectiveness in wound healing [71]. In mammalian anti-infection animal models, the antibacterial peptide FWKFK demonstrated strong antibacterial activity, suggesting that it may have use in wound healing [71]. An in vivo rat open wound model was employed to illustrate the usefulness of D-GW1 in wound healing, as it not only dramatically suppressed bacterial growth but also aided in wound healing and re-epithelialization [72]. Furthermore, the treatment group's regenerated granulation tissue thicknesses were significantly higher than those of the control group, providing additional evidence for D-GW1's effectiveness in accelerating wound healing [72]. AMP (RWPIL) has demonstrated promising properties against bacteria that are resistant to drugs on wounds, hence facilitating the healing process [62].

Wound-Healing Mechanism of Antimicrobial Peptide

**Figure 5:**
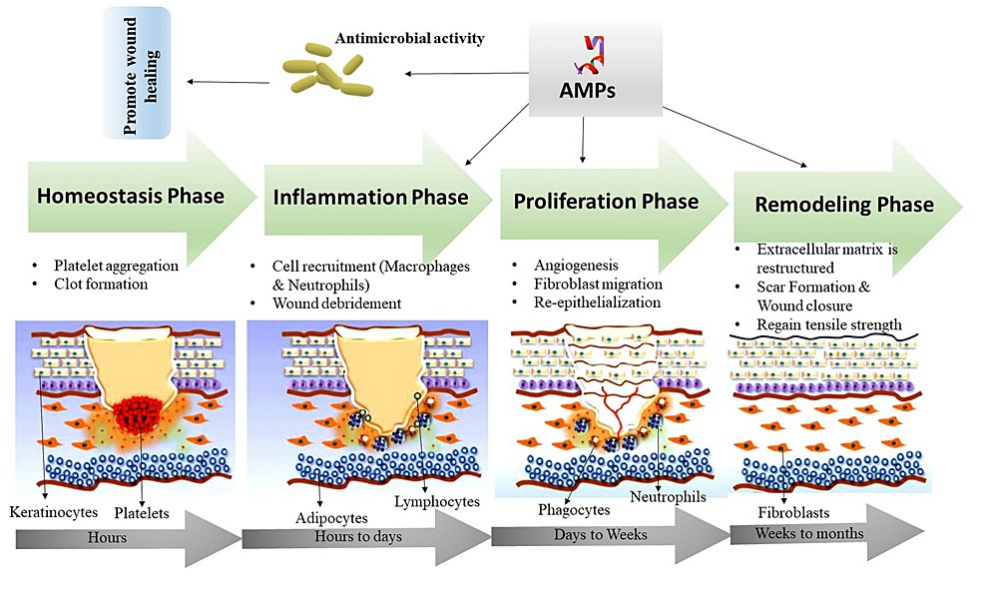
The mechanism of AMP in wound healing AMP: antimicrobial peptide Image Credit: Author

Wound healing is a multistep process that includes hemostasis, inflammation, proliferation, and remodeling. Figure 5 depicts four critical steps in the AMP wound-healing process: the hemostasis stage occurs when bleeding ceases because platelets compress together to form a clot [40,55]. During the inflammatory phase, immune cells, such as neutrophils and macrophages, migrate to the wound site to combat infection and remove debris [40]. During the proliferation stage, new tissue is formed by mechanisms such as angiogenesis, fibroblast migration (the movement of collagen-producing cells), and re-epithelialization [55]. During the tissue remodeling stage, collagen and other extracellular matrix components are deposited to help support and shape the new tissue [40,55].

One example of a peptide involved in wound healing is LL-37, a human cathelicidin AMP. LL-37 has been demonstrated to increase fibroblast collagen synthesis, which is necessary for tissue healing and wound closure. LL-37 can also increase the migration and proliferation of epidermal keratinocytes, which helps to regenerate the epidermis layer [15,16,20,21].

LL-37 acts as an immunomodulator during the inflammatory phase of wound healing, triggering bacterial lysis, increasing macrophage phagocytosis, and inhibiting infection spread. These acts aid in the clearance of infections and inflammation, hence promoting overall wound healing [15,16,20,21].

According to an emerging body of research, several antimicrobial drugs have a unique mechanism that aids in wound healing [55]. To help understand diverse methods, we have given a few examples of AMPs that aid in wound healing in this paper. Cathelicidin-DM wound healing involves a number of critical pathways and processes [55]. The study adds information on the potential routes via which cathelicidin-DM promotes wound healing [55].

Macrophages are necessary for wound healing, particularly during the inflammatory period [55]. Cathelicidin-DM has been shown to stimulate macrophage recruitment to the wound site, assisting in the elimination of wounded tissue and antigens while also enhancing cell proliferation, migration, and angiogenesis [55]. According to the study, cathelicidin-DM causes mouse RAW264.7 cells, a type of macrophage, to move in vitro [55]. Furthermore, cathelicidin-DM has been shown to stimulate the expression of macrophage-derived growth factors and chemokines [55]. It has also been shown that additional wound-healing peptides, such as salamander tylotoin and frog Tiger17, recruit macrophages to the wound site. As a result, one important way in which cathelicidin-DM promotes wound healing may be through macrophage recruitment [55].

Promotion of cell proliferation and migration: Cathelicidin-DM increases cell migration and proliferation, accelerating wound healing [55]. In vitro studies have demonstrated that cathelicidin-DM enhances the migration of RAW.264.7, HSF, and HUVEC cells while also enhancing the proliferation of certain cells [55]. Furthermore, cathelicidin-DM has been shown in mice to accelerate the creation of granulation tissue and re-epithelialization at the site of skin injury, suggesting its potential to aid in tissue regeneration and wound closure [55]. These results are explained by increased phosphorylation of ERK, JNK, and P38, which are involved in cell migration and proliferation, activating the MAPK signaling pathway [55].

Activation of MAPK signaling pathway: Cathelicidin-DM increases the MAPK signaling cascade by increasing the phosphorylation of proteins involved in cell migration, proliferation, and inflammation, such as ERK, JNK, and P38 [55].

Acceleration of skin wound healing: Cathelicidin-DM facilitated skin wound healing [45,55].

Immune regulation: Even though cathelicidin-DM did not influence the release of specific cytokines, it could nonetheless be essential to immunological control throughout the wound-healing process [45,55].

Bifunctional peptide activity: Cathelicidin-DM is a bifunctional peptide that can both heal wounds and fight bacteria, which makes it a good option for treating chronic wounds that are infected [45,55].

These findings show that cathelicidin-DM may have a variety of roles in the wound-healing process, allowing for a novel strategy for the treatment of chronically infected wounds [45,55]. To summarize, cathelicidin-DM has the potential to cure antibiotic resistance and chronic wound infections by accelerating skin wound healing, activating the MAPK signaling pathway, and promoting cell proliferation and migration [55].

The AMP SR-0379 stimulates the PI3 kinase-Akt-mTOR pathway, which speeds up wound closure and promotes healing [23]. This mechanism involves higher antibacterial activity against a wide range of bacteria, including drug-resistant strains, as well as improved angiogenesis, granulation tissue formation, and endothelial and fibroblast proliferation [23]. Furthermore, studies have shown that SR-0379 outperforms fibroblast growth factor 2 (FGF2) in diabetic and immunodeficient rat models, significantly speeding wound healing [23]. Studies on SR-0379 have shown that it improves wound healing. In lab experiments, SR-0379 has shown its ability to improve crucial activities such as tissue contraction, migration, tube creation, and cell proliferation, all of which are essential for healing skin wounds [23]. In vivo studies revealed that the treatment was also effective in encouraging wound closure by stimulating angiogenesis, granulation tissue growth, collagen production, and cell proliferation [23]. Notably, SR-0379 outperformed FGF2 in promoting rapid healing without infection in an ulcer model [23].

The synthetic peptide Pep19-2.5 may have therapeutic uses for wound healing as well as suppressing inflammatory and immunological responses in skin cells [38]. These are addressed in this study. The study investigates how Pep19-2.5 influences keratinocyte cell motility, ATP release, calcium mobilization, and mitochondrial ROS generation [38]. It also assesses Pep19-2.5's ability to accelerate wound healing in a mouse model and demonstrates how it does so [38]. Three components of the mechanism of action include intracellular calcium, ROS, and the P2X7 receptor [38].

**Figure 6:**
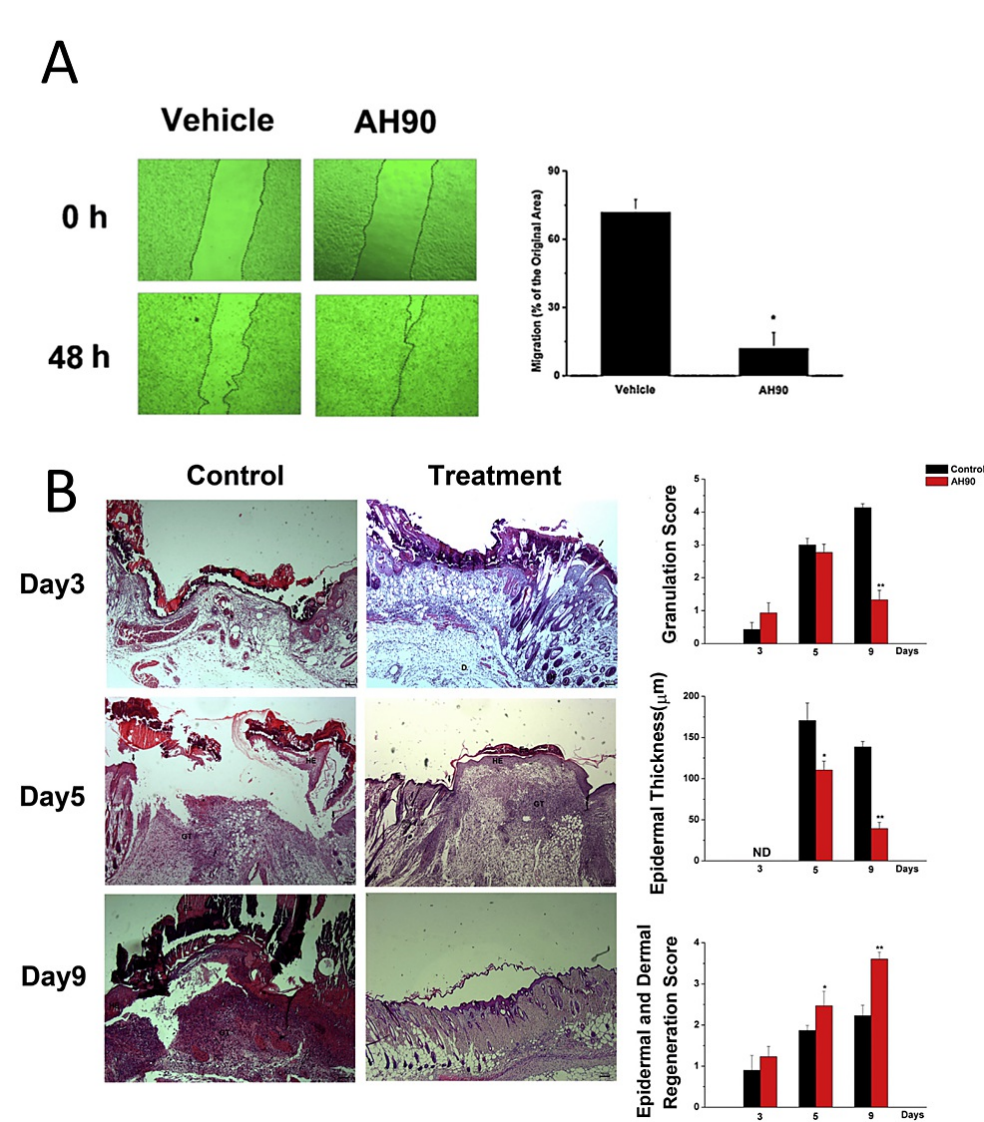
The mechanism of AH90 in wound healing: Panel A shows the effects of AH90 on HaCaT cell migration and their role in wound healing. Panel B depicts the histological analysis of the wound in mice Image Credit: Adapted with permission from Liu et al., 2014 [41]

One study paper discusses the unique cathelicidin peptide known as cathelicidin-NV, which is derived from the frog *Nanorana ventripunctata*'s skin secretions, and its potential wound-healing properties [40]. The peptide has been shown to promote wound healing by activating the MAPK signaling pathway, releasing cytokines and chemokines associated with wound healing, and increasing the proliferation and mobility of keratinocytes and fibroblasts. Cathelicidin-NV is an excellent choice for skin wound treatments since it has neither cytotoxicity nor antibacterial properties [40]. The article discusses the discovery of AH90, a peptide isolated from the skin of the frog *Odorrana grahami* that has the potential to improve wound healing [41]. By inducing the production of transforming growth factor-β1 (TGF-β1) and triggering the TGF-β/Smad signaling pathway, the peptide was seen to facilitate wound healing in mice [41]. Additionally, AH90 promoted cell adhesion to laminin and fibronectin, which aided in the healing of wounds [41]. As seen in Figure 6, AH90 demonstrated a noteworthy function in wound healing via the migration of HaCaT cells [41]. Figure 6A demonstrated that HaCaT cells moved inside and covered a larger area of the wound when AH90 was present, aiding in the healing process [41]. Additionally, figure 6B's histological investigation confirmed that AH90 AMP facilitates wound healing by encouraging dermal and epidermal regeneration, the creation and deformation of granulation tissue, and a drop in epidermal thickness [41].

By promoting wound closure and the proliferation of airway epithelial cells, among other processes, LL-37 aids in the healing of wounds [21]. Furthermore, LL-37 promotes angiogenesis, up-regulates VEGFa and IL-6 production, and performs immunomodulatory actions such as antibacterial activity and pro-inflammatory response regulation to expedite wound healing [20]. Moreover, it has been shown that LL-37 functions as a chemoattractant, causing leukocytes to infiltrate the wound and encouraging keratinocyte migration-a process crucial to wound healing. Research has demonstrated that histatin-2 can promote fibroblast migration, a critical phase in the healing of wounds, without causing inflammation or the formation of fibrosis [14]. This implies that histatin-2 contributes to wound healing without inducing fibrosis or unfavorable inflammatory reactions [14].

In the end, through a variety of processes, AMPs are essential for wound healing. These peptides support the creation of clots to halt bleeding, which helps to achieve hemostasis [64]. AMPs draw immune cells to the wound site, such as macrophages and neutrophils, to combat infection and clear debris [69]. Furthermore, AMPs have antibacterial qualities that lower the chance of infection and hasten the healing of wounds [60]. The process of wound healing is intricate and dynamic, requiring the coordinated actions of many cells, extracellular matrices, and cytokines to repair tissue and function appropriately [26,54].

Safety and Toxicity Considerations

LL-37's toxicity and safety have been thoroughly investigated [15]. Research has demonstrated that LL-37 can be cytotoxic to cultivated HCECs at concentrations greater than 10 µg/mL [15]. Additionally, the rapid breakdown of LL-37 in the wound environment makes it difficult to employ therapeutically; instead, large dosages and frequent dosing are required, as are alternative delivery strategies such as gene therapy [20]. Furthermore, while evaluating the potential of LL-37 for therapeutic uses, it is critical to consider its cytotoxic effects at higher dosages. These findings underline the need to understand the LL-37 profile's safety and toxicity before using it in clinical settings [20]. Another investigation found that human microvascular endothelial cells (HMECs) were used to assess the cytotoxicity of LL-37 [20]. The results showed that recombinant P-LL37 had no effect on HMEC viability at any of the investigated doses, and at certain concentrations, it even modestly improved vitality [16]. Furthermore, when LL-37 was applied topically every day for seven days in wound-healing experiments on dexamethasone-treated mice, no side effects were seen [16].

Numerous studies have assessed the toxicity and safety of PXL150 [24]. When PXL150 was given systemically to rats in a repeated dosage toxicity trial, there was no treatment-related mortality or systemic toxicity seen [24]. Furthermore, PXL150 caused neither ocular nor cutaneous toxicity in a rabbit local tolerance test, and no treatment-related systemic toxicity was noted [24]. These results bolster the safety of PXL150 for possible usage in wound-healing applications [24].

To assess the toxicity of TP3 in mice, intramuscular bolus injections in the left thigh at a dosage of two milligrams per mouse were used [25]. Blood samples were taken on days 1, 3, and 6 following the last injection to measure the serum levels of several biochemical markers, and the mice were attentively watched for indications of systemic toxicity [25]. Utilizing the Baby Hamster Kidney cell line for in vitro toxicity investigations, it was demonstrated that TP3 did not affect cell viability at doses as high as 40 μg/ml [25]. Furthermore, even at very high doses (2 mg/mouse), an acute toxicity test on mice showed that TP3 did not result in significant adverse effects within 60 minutes of exposure [25]. The fact that TP3 is not immunotoxic and that it is compatible with antibiotics lends more credence to its safety profile. Because of its preventive effectiveness and lack of capacity to cause resistance, TP3 has been considered a useful addition to the usage of antibiotics and to be appropriate in high-risk infection scenarios [25]. Overall, the results show that TP3 is a good candidate for additional research as an antibacterial agent since it does not cause systemic toxic effects in mice and does not exhibit acute toxic effects [25]. The lack of appreciable alterations in blood biochemical parameters suggests that TP4 has been demonstrated to have minimal toxicity in mice [26]. Furthermore, human fibroblast and keratinocyte cell lines showed no harmful effects from TP4, and in fact, the proliferative activity of these cell lines increased. According to these results, even at high doses, TP4 appears to be safe and well-tolerated, with no discernible systemic adverse effects [26].

The peptide 120-146 WH generated from CAP37 showed less cytotoxicity at lower dosages, according to the research findings [27]. This implies a good safety profile for possible therapeutic uses in the management of wound healing and bacterial infections [27]. To properly evaluate its safety and toxicity profile, more research would be required, particularly at greater doses or in other biological systems [27]. It has been demonstrated that AMPs, such as Cys-KR12, may be cytotoxic and susceptible to proteolysis [31]. Immobilization strategies have been used to decrease cytotoxicity and boost stability in a physiological setting to address these problems [31].

Myxinidin2 and myxinidin3 did not exhibit any cytotoxicity at 50 μM in cytotoxicity tests conducted on HaCaT cells, suggesting that skin cells may safely use them [32]. Furthermore, myxinidin2 failed to display any toxicity when assessed for hemolysis at an amount up to 50 μM, but myxinidin3 exhibited 17% hemolysis at the same quantity [32]. These results point to the possible safety of myxinidin2 and myxinidin3 for therapeutic application by revealing their minimal cytotoxicity and hemolytic activity at effective antibiotic doses [32]. Through a variety of tests, the toxicity and safety of cathelicidin-NV were assessed. When assessed for cytotoxicity against a variety of cells, cathelicidin-NV was found to be non-cytotoxic [40]. Furthermore, hemolytic tests employing suspensions of rabbit erythrocytes revealed no hemolytic activity for cathelicidin-NV [40]. According to these results, cathelicidin-NV may be safe for use in therapeutic settings due to its minimal cytotoxicity and hemolytic potential [40]. After examining the safety and toxicity aspects of Epi-1, researchers discovered that the viability of HaCaT cells was unaffected by certain doses of Epi-1 [33]. Furthermore, it has been demonstrated that Epi-1 stimulates the migration and proliferation of epithelial cells in vitro without causing cytotoxicity [33]. According to these results, Epi-1 appears to have a minimal toxicity profile and a positive safety profile at effective doses [33].

In animal investigations, the toxicity and safety of cathelicidin-OA1 were assessed. After two weeks, mice injected with cathelicidin-OA1 at doses of 5, 10, 20, and 40 μmol/kg exhibited no fatal effects or aberrant behaviors, indicating the drug's safety potential for therapeutic use [36]. According to research by Mi et al. (2018), A-hBD-2 had fewer cytotoxic effects than hBD-2 and did not cause any harm to HaCaT cells at doses ranging from 0 to 100 μg/mL [37]. According to the study, peptides KR-12 and VQ-12 V26 showed less cytotoxicity to HaCaT cells and human erythrocytes, suggesting that they would be safe for use in wound-healing applications [39]. Furthermore, the safety profile of peptides was reinforced by the fact that they did not cause any harm to epithelial cells [39].

An in vitro biocompatibility test was conducted to assess the biocompatibility of Tet213-immobilized wound dressings. The findings demonstrated good vitality and proliferation of cells cultivated on the dressings [42]. Furthermore, the CCK8 colorimetric assay and Live/Dead staining revealed no discernible cell death or unfavorable effects on the cells, indicating the inherent biocompatibility of the composite dressings [42]. Even though these investigations show that Tet213 is biocompatible, more investigation may be required to completely determine the safety and toxicity of Tet213 [42].

Except for a little increase in ALT levels in 14% of the mice, which may have been brought on by partial hemolysis in the blood samples, CAMP-A has been demonstrated to have no harmful effects on experimental mice [43]. Furthermore, the mice given CAMP-A treatments did not experience any changes in their body weight, behavior, or hematological markers. On the other hand, a small number of mice showed raised ALT levels in response to greater dosages of CAMP-A, suggesting possible liver injury [43]. To completely comprehend the safety profile and any harmful consequences of CAMP-A, further research is required [43]. Most people agree that histatin-1 is safe and non-toxic. This salivary peptide is a naturally occurring substance that has been researched for possible medical uses, such as wound healing. But safety and toxicity concerns are crucial, just like with any prospective medicinal drug. There hasn't been much research done on the safety of histatin-1 in this research article, and further research is required to completely grasp its safety profile [46].

KK(SLKL)3KK was tested for toxicity in vitro on NIH-3T3 fibroblasts. NIH-3T3 fibroblasts did not die when exposed to AMP-HA hydrogel solutions at concentrations between 10 and 1000 μg/mL, with statistically significant differences [48]. Furthermore, in vivo biosafety investigations revealed that the AMP-HA hydrogel did not significantly impair liver or renal function or cause systemic toxicity [48]. These results imply that there is no discernible in vivo or in vitro toxicity associated with KK(SLKL)3KK [48]. Using the XTT test on the HEK-293 cell line, the cytotoxicity of DCD-1L was assessed. It was discovered that DCD-1L had no discernible harmful impact on cell viability [51]. Furthermore, at the same dosages, DCD-1L showed no hemolytic action against red blood cells [51]. These results suggest that DCD-1L is both hemocompatible and safe [51].

It has been noted that TP2-5 and TP2-6 encourage cell migration and proliferation in a variety of cell types while having little cytotoxicity [53]. According to these results, TP2-5 and TP2-6 may be safe to employ in wound-healing applications due to their low toxicity [53]. In one study, the cytotoxicity of LL-37 was assessed using a PU-adhesive-LL37NP dressing [56]. The results indicated that the dressing was not cytotoxic to keratinocytes [54]. Furthermore, the dressing demonstrated strong cytocompatibility with fibroblasts and keratinocytes, two types of skin cells [54]. The HELF and 3T3-L1 cells were used in the toxicity experiment to evaluate the toxicity of Pt5-1c [56]. The results indicated that Pt5-1c, even at a high dosage, exhibited very low toxicity against 3T3-L1 cells but minimal toxicity toward HELF cells [56].

According to Xu et al. (2022), C-At5 is biocompatible and has less toxicity when combined with AuNRs. AuNR@C-At5 exhibited a hemolysis rate of less than 5% in human red blood cells, suggesting a high degree of compatibility with blood cells [57]. Conversely, at 80 μg/mL, AuNR@CTAB itself had a 60% hemolysis rate, making it extremely hazardous [57]. Furthermore, at low concentrations, it was non-toxic to HFF-1 cells, demonstrating the cytocompatibility of AuNR@C-At5 [57]. Additionally, the study discovered that conjugating the peptides to AuNRs greatly decreased the toxicity of C-At5, suggesting a decrease in cytotoxicity against human cells [57]. Overall, the work supports C-At5's potential for usage in wound-healing applications by demonstrating its high biocompatibility and decreased toxicity when coupled to AuNRs [57]. Many studies evaluated the toxicity of Esc(1-21) and Esc(1-21)-1c. After 24 hours of peptide treatment, Esc(1-21)-1c was shown to be non-toxic, with 100% cell survival [60]. Furthermore, prior research has shown that Esc(1-21) and Esc(1-21)-1c effectively reduced bacterial corneal infection without exhibiting any harmful side effects [60]. Moreover, Esc(1-21)-1c was shown to be non-toxic in mouse research where it was administered at certain doses without causing any negative side effects [60].

Testing on hydrogels containing the AMP HX-12C has shown that it is not very harmful, as evidenced by studies on hemocompatibility and cytotoxicity [61]. With cell survival exceeding 90%, the hydrogels were shown to have high biocompatibility and to be almost non-cytotoxic to L929 cells [61]. Additionally, the hemolysis rate was 5% below the national safety standard for biological materials [61]. Furthermore, it has been demonstrated that encasing AMPs in hydrogels effectively lessens their cytotoxicity [61].

Using the mouse embryonic fibroblast cell line NIH-3T3, the cytotoxicity of the C8G2 peptide was assessed [64]. The findings demonstrated that cell survival following treatment with 1.25% C8G2/BGA hydrogel was greater than 90%, suggesting minimal cytotoxicity [64]. Cell viability dropped to less than 85% when the peptide content was raised to 1.5% due to the hydrogel's enhanced cytotoxicity [64]. These results imply that the concentration of C8G2 in the hydrogel can have an impact on its cytotoxicity. Actinomycin X2 has been shown to be much less cytotoxic when immobilized in ASF film as opposed to when it is free [67]. The structure of free Ac. X2 may facilitate cell membrane perforation, and it can intercalate into duplex DNA to impede DNA-dependent RNA polymerase activity and protein synthesis, which accounts for its high cytotoxicity [67]. Because of its decreased cytotoxicity, ASF film is a viable option for applications involving wound healing [67].

At dosages of 16 μg/mL, PG1 reduces the migration rate and may cause cell toxicity, indicating a risk at larger concentrations [68]. Furthermore, it has been postulated that the lack of active migration of PG1 Tg KCs might be attributable to PG1 toxicity [68]. These findings imply that, while PG1 may be useful in wound healing, clinical applications should carefully consider its concentration and possible toxicity [68]. A study on the toxicity of FWKFK revealed that the AMP was highly cell-compatible and caused almost little harm to normal cells [71]. Furthermore, FWKFK was proven in the study to have no hemolytic effect on red blood cells, indicating that it is safe for use in wound-healing applications [71]. Furthermore, the study found that FWKFK was safe to use in improving wound healing in animal models since it had no negative effects on tissue or organs [71]. These findings suggest that FWKFK is both safe to employ in wound-healing applications and low in toxicity. According to Zhou et al. (2023), Jelleine-1 exhibited little toxicity both in vivo and in vitro [73].

Numerous studies did not particularly address the toxicity and safety issues related to certain AMPs [15,24]. It is critical to recognize that, for any drug delivery system or wound-healing application, safety and toxicity assessments are essential parts of the review procedure [36,40]. These assessments usually involve studies on cytotoxicity, acute and chronic toxicity, and potential adverse effects on the target tissues or organs [16,25].

In light of the information at hand, it would be imperative that AMPs undergo a comprehensive assessment for toxicity and safety in further studies, particularly in relation to their potential use as a medicine for wound healing [20,27,33]. In-depth in vitro and in vivo research would be needed to assess the potential cytotoxic effects, systemic toxicity, and any adverse reactions related to the use of AMPs in wound-healing applications [24,28,48]. Additionally, it would be beneficial for researchers to investigate potential systemic distribution and long-term effects in order to ensure that AMPs are safe for use in clinical settings [71,73]. Careful evaluation of these parameters is necessary for the development of effective and safe wound-healing therapies [71,73].

Limitations of this study

There are some limitations to this systematic review paper. The study's coverage of wound-healing applications and the number of AMPs may be restricted. To get more thorough insights, a wider spectrum of AMPs and wound-healing situations must be taken into account. Because only a subset of research publications are included in the study, publication bias may be present. The evaluation of AMPs' overall safety and effectiveness in wound healing may be impacted by this bias.

## Conclusions

The research presented in this article highlights the significant potential of several AMPs in promoting faster wound closure, reducing inflammation, enhancing collagen deposition, stimulating angiogenesis, and inhibiting bacterial growth. These AMPs have demonstrated encouraging outcomes in both in vitro and in vivo investigations, implying that they can hasten the healing of wounds and fend off infections.

In addition to their antimicrobial properties, AMPs have emerged as valuable agents in wound healing due to their multifaceted mechanisms of action. The diverse range of AMPs discussed in this article, each with unique characteristics and biological activities, offers a rich landscape for further exploration and development in the field of wound healing. Harnessing the power of AMPs in combination with advanced delivery systems and biomaterials holds promise for improving clinical outcomes and addressing the challenges associated with non-healing wounds. Continued research efforts aimed at elucidating the molecular pathways and optimizing the formulations of AMP-based therapies are essential for realizing the full therapeutic potential of these bioactive peptides in the context of wound management and tissue regeneration.
